# Metasurface-based large field-of-view light receiver for enhanced LiDAR systems

**DOI:** 10.1515/nanoph-2025-0161

**Published:** 2025-07-02

**Authors:** Hanwen Guo, Xiangkun Zhou, Bo Gao, Jianing Yang, Lingyun Zhang, Junya Wang, Zheng You

**Affiliations:** Microsystems Technology Research Center, School of Mechanical Science and Engineering, 12443 Huazhong University of Science and Technology, Wuhan, 430074, China; State Key Laboratory of Digital Manufacturing Equipment and Technology, 12443 Huazhong University of Science and Technology, Wuhan, 430074, China

**Keywords:** metasurface, near-infrared, LiDAR, receiving optical system

## Abstract

This paper presents a metasurface-based light receiver tailored for compact off-axis light detection and ranging (LiDAR) systems, addressing the critical challenge of simultaneously enhancing the field of view (FOV) and effective signal reception while adhering to strict size and weight limitations. A general design principle for the metasurface-based light receiver with large FOV capability is proposed, leveraging mapping relations to achieve optimal performance. As a proof of concept, a 20-mm-diameter 4-region metasurface device was designed and fabricated by deep ultraviolet (DUV) projection stepper lithography on an 8-inch fused silica wafer. The metasurface-based light receiver achieves a large FOV of ±30° and demonstrates a significant power enhancement ranging from 1.5 to 3 times at 940 nm when coupled with a 3-mm-diameter avalanche photodiode (APD). The innovation not only establishes a new paradigm for compact, high-performance LiDAR systems but also enables deployment in advanced fields such as unmanned aerial vehicles (UAVs) and miniaturized robots.

## 1 Introduction

Light detection and ranging (LiDAR) is a high-speed and high-accuracy technology for acquiring three-dimensional information, widely applied in fields such as self-driving cars [1], unmanned aerial vehicles (UAVs) [2], environmental monitoring [3] and ocean exploration [4]. A typical LiDAR system comprises two main components: the transmitting system and the receiving system. The transmitting system scans the target by dynamically deflecting a laser beam. The receiving system, which integrates both optical elements and detectors, is responsible for capturing the echo light signals reflected by the target. To enable LiDAR systems to receive weak signals with small size and low power consumption, avalanche photodiode (APD) [5], [6], [7] and single photon avalanche diode (SPAD) [8], [9] with single-photon detection capability are commonly employed as detectors. The detectors rely on the optical elements capable of providing a large field of view (FOV) and enhancing the strength of the received signals to increase the detection range and signal-to-noise ratio (SNR). Conventional optical configurations, composed of lenses and auxiliary optical elements such as fiber optic taper and immersion lens, can expand the FOV while maintaining the focusing capability. However, these configurations are bulky, heavy, difficult to install and limited in increasing the FOV, making them unsuitable for lightweight and compact LiDAR systems with large FOVs [10]. Although some LiDAR systems utilizing APD arrays to enlarge the FOV have been proposed [11], [12], [13], these arrays involve complex manufacturing processes, significant costs, and still result in relatively large system volumes. Therefore, there remains a strong demand for a compact light receiver with a large FOV using a single detector.

Metasurfaces, as a novel class of diffractive optical elements, use subwavelength nanopillars arranged periodically on a thin substrate to manipulate the phase, amplitude and polarization of incident light [14]. Compared to conventional optical elements, metasurfaces offer superior advantages due to their lightweight, ultrathin nature, flexibility in design and compatibility with semiconductor processes [15]. Metasurfaces have been extensively studied for imaging and beamforming applications [16]. In LiDAR transmitting systems, for example, metasurfaces have been used for transmissive beam steering and deflection angle enlarging [17], [18], [19]. However, the exploration of metasurfaces in non-imaging scenarios, such as light receiving, remains limited [20], [21]. The light receiver of a LiDAR system requires the metasurface to achieve signal enhancement across a large FOV. While the large FOV metasurfaces have been investigated using various methods, such as cascaded metasurface [22], metasurface array [23], and quadratic metasurface [24], [25], [26], almost all of them are utilized for imaging applications like palm print recognition [27], fingerprint recognition [28] and wide-angle stereo imaging [29].

Currently, the primary fabrication method for metasurface involves electron-beam (e-beam) lithography [30], ensuring a nanoscale patterning resolution. However, this kind of point-by-point techniques is time-consuming, expensive and not well-suited for mass production. As the diameter increases, the number of nanopillars grows significantly, making it challenging to fabricate large-aperture metasurfaces. In recent years, several viable fabrication methods for large-aperture metasurface have been proposed, such as DUV projection lithography [31], [32], [33], nanoimprint lithography (NIL) [34] and e-beam lithography based on the variable shaped beam (VSB) and character projection (CP) writing principle [35]. The maximum exposure area of DUV projection lithography is approximately 20–30 mm, allowing a metasurface with a 20 mm diameter to be conveniently fabricated in a single exposure.

In this study, a large FOV metasurface-based light receiver for LiDAR systems is systematically investigated. Compared to conventional receiving systems, the metasurface-based light receiver has the capacity to significantly reduce the weight and size of the LiDAR system, thereby attaining a compact footprint. This paper first elucidates the design principle for the metasurface-based light receiver with large FOV capability, which is grounded in mapping relationships. It then introduces a metasurface fabrication method based on the DUV projection stepper lithography technique to satisfy the requirement for batch production of large-aperture metasurfaces. Subsequently, a 20-mm-diameter 4-region metasurface device with a ±30° FOV at 940 nm was designed and fabricated. The metasurface device can converge the echo signal onto a 3-mm-diameter APD located 15 mm away from the metasurface plane. The performance of the metasurface was experimentally evaluated, confirming a power enhancement ranging from 1.5 to 3 times. Finally, through the 3D imaging experiment conducted with a microelectromechanical system (MEMS) LiDAR, the feasibility of metasurface in practical applications is demonstrated.

## 2 Design and fabrication of the metasurface

### 2.1 Design

**Figure 1:**
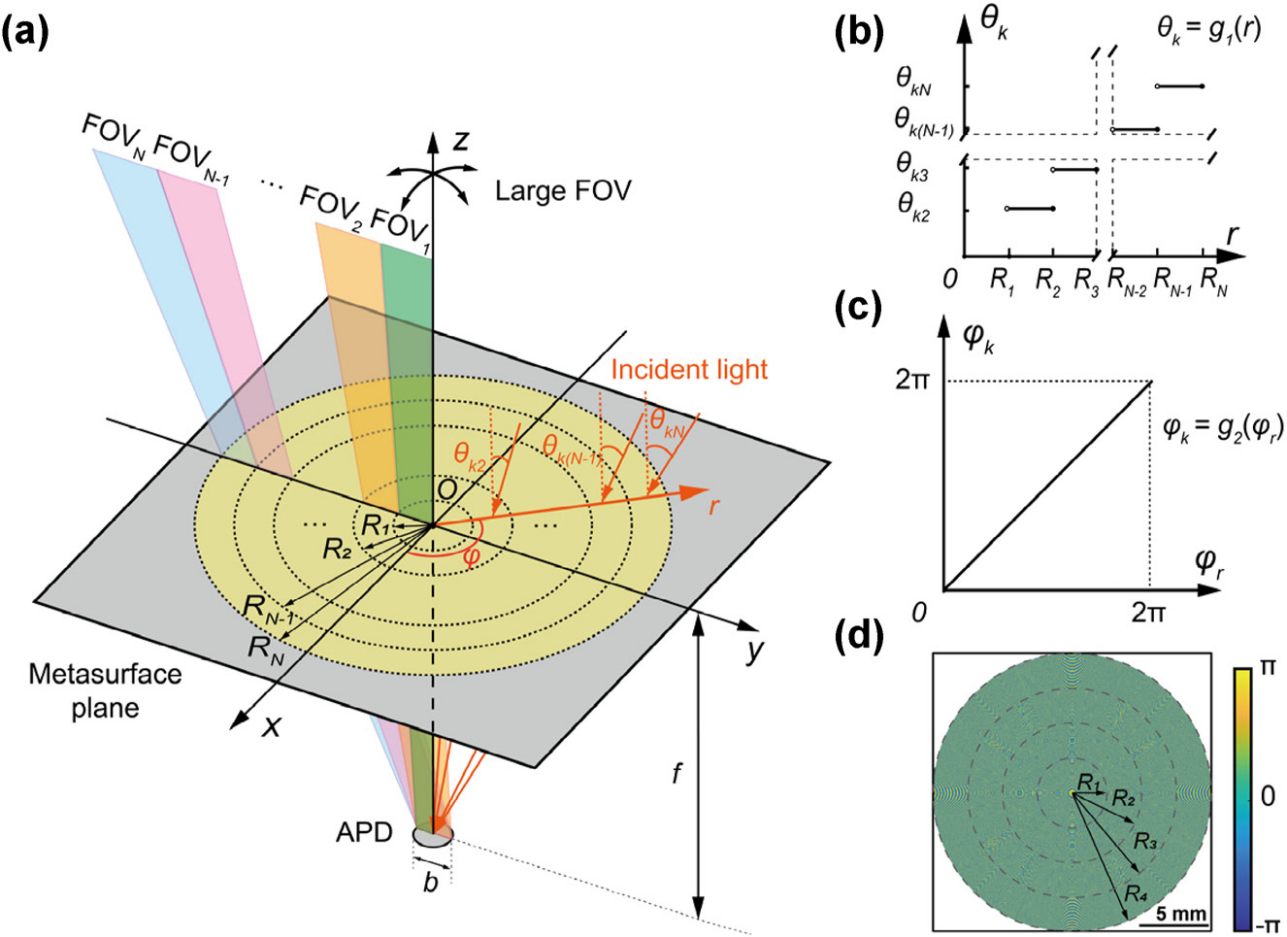
Working principles of the metasurface-based light receiver. (a) Schematic diagram of the metasurface-based light receiver with a large field of view (FOV). (b) Graph of the designed staircase function *g*_1_(*r*). (c) Graph of the designed function *g*_2_(*φ*_*r*_). (d) Phase profile of the 20-mm-diameter 4-region metasurface device.

A general design methodology of the non-imaging metasurface for large FOV light receiving is elaborated in this subsection, as shown in Figure 1. The light receiver consists of an optical metasurface and an APD detector with a diameter of *b* (Figure 1a). The metasurface device, placed in the *xOy* plane, ensures efficient light signal reception by the detector under various incident angles across the entire targeted FOV. To begin with, we assume that the metasurface converges a normally incident plane wave into a point with a focusing phase profile *Φ*(*x*, *y*). To maintain the focal position unchanged under oblique incidence compared to normal incidence, the metasurface is further imparted with a linear phase compensation, and the total phase profile can be expressed as:

(1) $$\Phi^{\prime }\left(x,y\right)=\Phi \left(x,y\right)+\frac{2\pi }{\lambda }\hat{k}\cdot r$$

where *λ* is working wavelength, ***r*** = (*x*, *y*) = *r* (cos*φ*_*r*_, sin*φ*_*r*_) is the position vector on the metasurface plane, $\hat{k}$ = (sin*θ*_*k*_ cos*φ*_*k*_, sin*θ*_*k*_ sin*φ*_*k*_, cos*θ*_*k*_) is the normalized incident wave vector, *θ* is the polar angle and *φ* is the azimuth angle in the spherical coordinate system. In this sense, $\hat{k}$ is a predefined constant and irrelevant to the position vector ***r***.

Subsequently, we consider the light receiving metasurface capable of directing beams from various directions to the original focal point simultaneously. In this scenario, *θ*_*k*_ can take any value within [0, *θ*_max_], and *φ*_*k*_ can take any value within [0, 2*π*], where ±*θ*_max_ represents the targeted FOV. Accordingly, *r* ∈ [0, *R*] and *φ*_*r*_ ∈ [0, 2*π*], where *R* denotes the radius of the metasurface. The design strategy involves ensuring that each incident direction $\hat{k}$ corresponds to a specific region on the metasurface, and a surjective mapping *g*: {*r*, *φ*_*r*_} ↠ {*θ*_*k*_, *φ*_*k*_} should be constructed. The surjectivity of *g* guarantees that every incident vector $\hat{k}$ corresponds to an output light energy distribution which covers the original focal point. For a practical detector with a finite photosensitive area rather than a geometric point, the surjective condition can be appropriately relaxed. For the sake of simplicity, the functional relationships between *θ*_*k*_ and *r*, as well as *φ*_*k*_ and *φ*_*r*_, can be established independently to avoid complex interrelations, which is *θ*_*k*_ = *g*_1_(*r*) and *φ*_*k*_ = *g*_2_(*φ*_*r*_).

As an example to demonstrate the proposed methodology, a quadratic phase profile is selected for focusing and *g*_1_(*r*) is chosen as a staircase function. The metasurface is designed as (Figure 1b and c):

(2) $$\left\{\begin{array}{c} \Phi \left(r\right)=-\frac{\pi r^{2}}{\lambda f}\;\\ \theta_{k}=g_{1}\left(r\right)=\theta_{\mathrm{kn}},\;R_{n-1}<r\leq R_{n}\;\\ \varphi_{k}=g_{2}\left(\varphi_{r}\right)=\varphi_{r}\; \end{array}\right.$$

where *f* is the focal length, *n* (= 1, 2, …, *N*) indexes the distinct segments of *g*_1_(*r*), *R*_*n*_ represents the boundary of the segmented ring regions, and *θ*_*kn*_ is a pre-defined constant. By this design, the metasurface device is divided into *N* regions and each ring region is responsible for receiving signals within a specific sub-FOV, denoted as FOV_*n*_. The overall FOV can be achieved by integrating the individual FOV_*n*_ of each region.

To obtain appropriate *θ*_*kn*_ values, more properties of the design should be analyzed. The quadratic phase profile converts the change in the incident angle of the beam into a horizontal displacement of the focal point in the focal plane as follows:

(3) $$\begin{array}{ll} \phi \left(x,y\right) & =\Phi \left(x,y\right)-k_{0}x\sin\alpha \\ & =-\frac{k_{0}}{2f}\left[{\left(x+f\sin\alpha \right)}^{2}+y^{2}\right]+\frac{k_{0}f\sin^{2}\alpha }{2} \end{array}$$

where *α* is the incident angle of the beam, *ϕ*(*x*, *y*) is the phase distribution of the outgoing light. Since the last term has no relation to variables and doesn’t affect the wavefront, each incident angle corresponds to a focus in the focal plane with an in-plane translation of *f*·sin*α*. For the detector with a diameter of *b*, the maximum incident angle to maintain the focal position within the photosensitive area is *α*_max_ = arcsin (*b*/2*f*). Hence, the staircase function *g*_1_(*r*) can be detailed as:

(4) $$g_{1}\left(r\right)=\theta_{kn}=2\left(n-1\right)\cdot \alpha_{\max},\;R_{n-1}<r\leq R_{n}$$

In this case, *θ*_*kn*_ is the center angle of the specific FOV_*n*_.

Finally, the metasurface phase profile can be expressed according to Eq. (1):

(5) $$\begin{array}{ll} {\Phi^{\prime }}_{n}\left(r\right) & =-\frac{k_{0}r^{2}}{2f}+k_{0}r\sin\theta_{kn},\;R_{n-1}<r\leq R_{n},\\ n & =1,2,\ldots ,N \end{array}$$

By this design, each region of the metasurface can receive the echo signal with an incident angle range from (*θ*_*kn*_ – *α*_max_) to (*θ*_*kn*_ + *α*_max_), the focus offset equals to *f*·(sin*θ*_*kn*_ – sin*α*) based on Eq. (3) and the focal position is in the center of the detector when *α* equals to *θ*_*kn*_. The phase profile has rotational symmetry about point O, the beams with incident angles ranging from –(*θ*_*kN*_ + *α*_max_) to (*θ*_*kN*_ + *α*_max_) can be received by the detector. Note that we chose quadratic phase profile for the sake of simplicity, but similar discussion can be conducted for other function form (hyperbolic phase profile, for example) if Taylor expansion formula is applied.

The specific phase profile is determined by the APD diameter *b*, the FOV of the light receiver, the distance *f* between the metasurface and detector, and the boundary radius *R*_*n*_ according to Eq. (5). As a proof of concept, a 20-mm-diameter 4-region metasurface device was designed, and the phase profile is shown in Figure 1d. The device is expected to direct all echo light signals within a FOV of about ±30° onto the effective photosensitive area of a 3-mm-diameter APD. The APD is positioned 15 mm away from the plane of the metasurface device.

**Figure 2:**
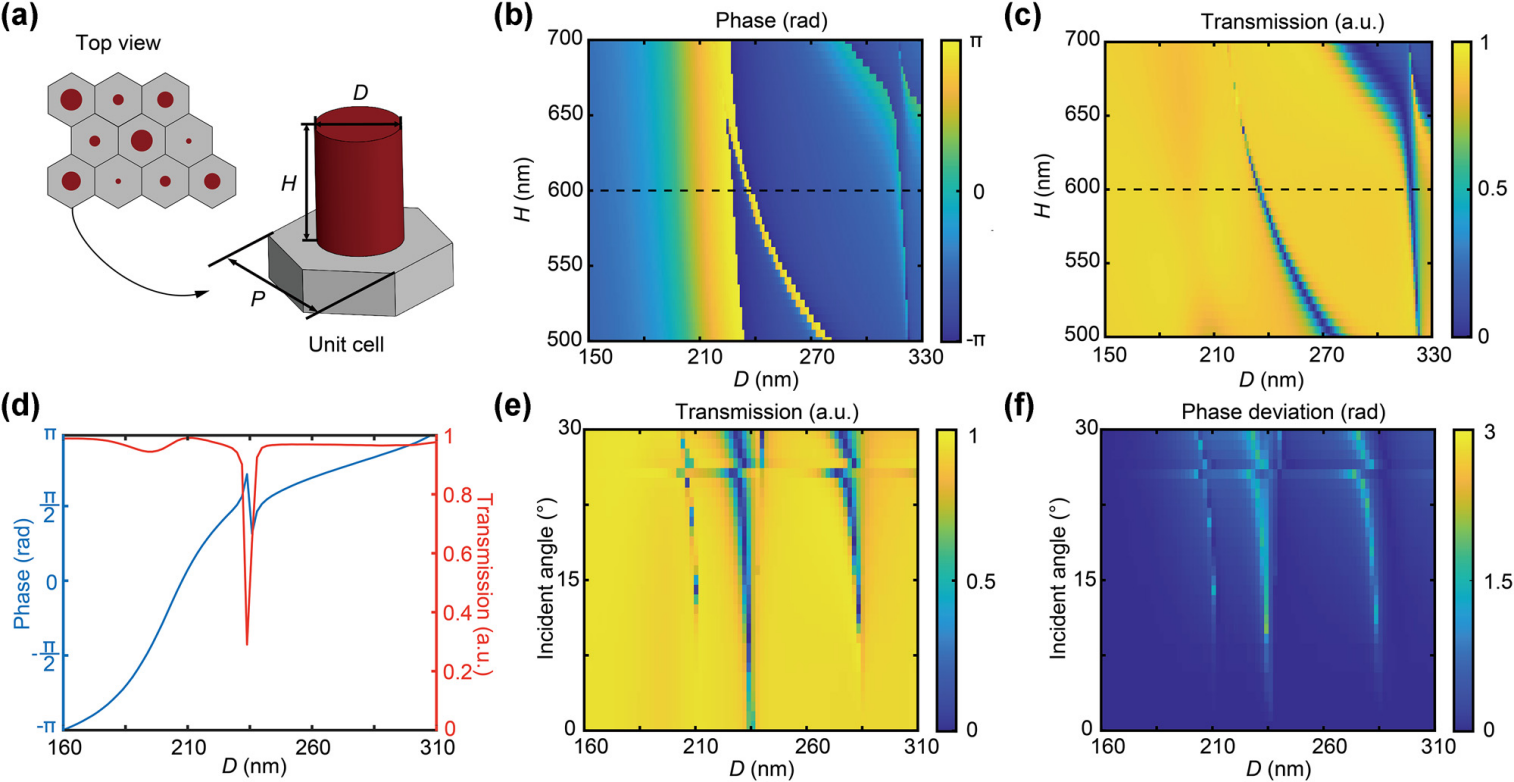
Design and simulation of the metasurface unit cell. (a) Schematic diagram of a single metasurface unit cell with period *P*, including an amorphous silicon cylinder of diameter *D* and height *H* on a fused silica substrate. (b) The transmissive phase and (c) transmission values of the unit cells as functions of *D* and *H* with a fixed period *P* = 450 nm at the wavelength of 940 nm. (d) The phase and transmission responses of the unit cell for *H* = 600 nm across varying *Ds*. (e) Angle-dependent optical power transmission of the cylindrical meta-atoms for different *Ds* and different oblique incident angles. (f) The deviation in the phase of the meta-atoms under oblique incidence compared to normal incidence.

The designed metasurface phase profile was then implemented by periodically arranged subwavelength unit cells, as shown in Figure 2. Each unit cell comprises an amorphous silicon cylinder placed on a fused silica substrate (Figure 2a), which is insensitive to the polarization state of the incident light. A hexagonal lattice arrangement is adopted to enhance the sampling resolution of the phase profile. For the working wavelength of 940 nm, the period *P* is set to 450 nm according to the Nyquist sampling criteria. The transmission coefficients can be modulated by varying the diameter (*D*) and height (*H*) of the nanocylinder (Figure 2b and c). The transmissive phase and transmission values are calculated using the finite-difference time-domain (FDTD) method, with *H* ranging from 500 to 700 nm and *D* ranging from 150 to 330 nm. Considering both the fabrication feasibility and the transmission modulation capability, *H* is fixed at 600 nm, ensuring an easily attainable aspect ratio. And the transmissive phase fully covers the range from −*π* to *π* while maintaining the transmission above 95 % by adjusting *D* from 160 to 320 nm (Figure 2d). To verify the incident-angle-insensitivity of the cylindrical unit cell design, additional numerical simulations were performed, and the results are presented in Figure 2e and f, which are detailed in Section S1 (Supplementary Material).

### 2.2 Fabrication

**Figure 3:**
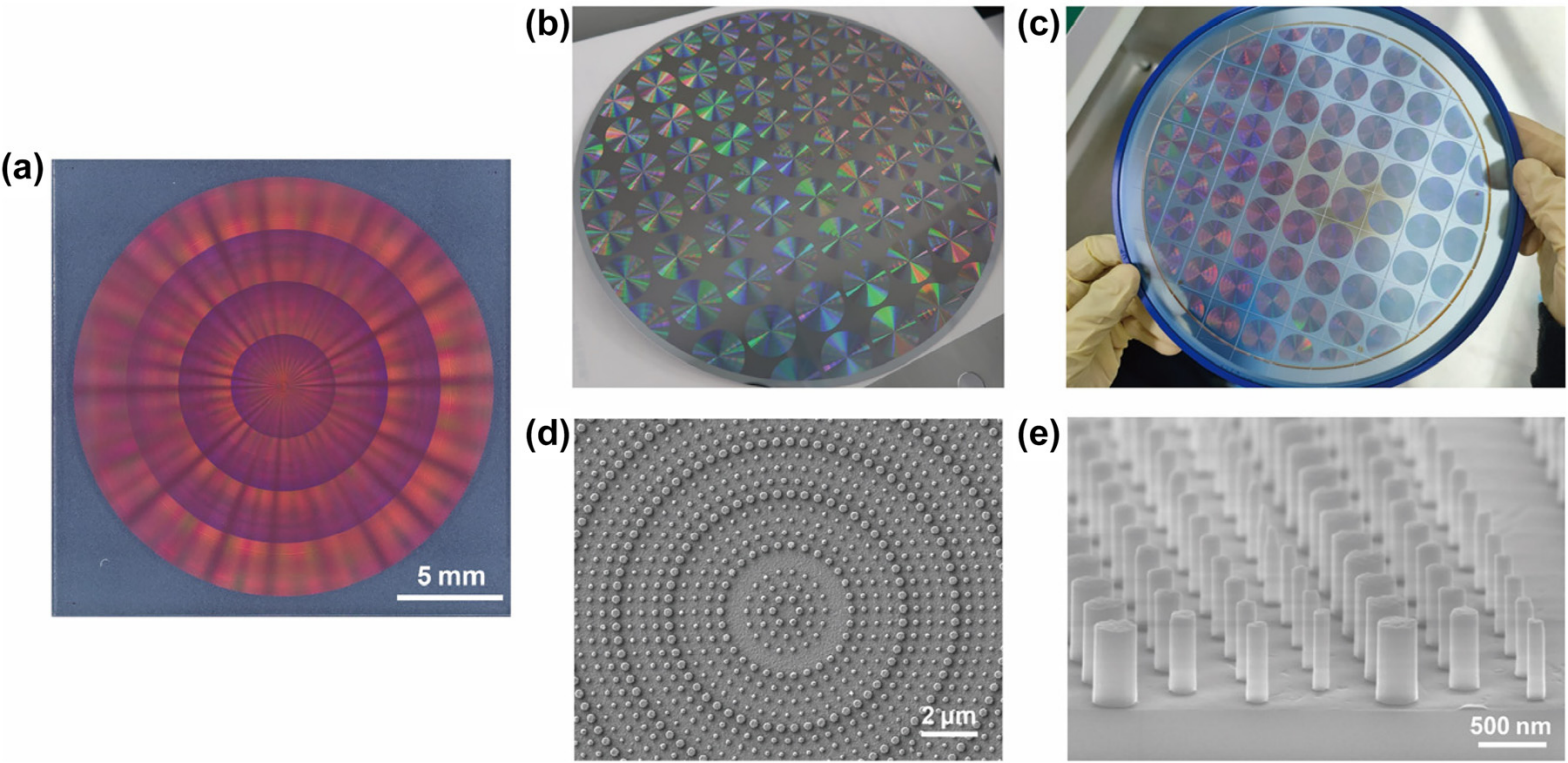
Images of the fabricated metasurface devices. (a) An optical microscopic image of one of the metasurface devices. (b) The entire 8-inch wafer before the etching process. (c) The entire 8-inch wafer after blade dicing. (d, e) Scanning electron microscopy (SEM) images of amorphous silicon nanopillars constitute the metasurface.

To meet the requirement for low-cost batch production, a high-throughput DUV stepper photolithography process was developed to fabricate 45 metasurface devices with a diameter of 20 mm on a single 8-inch fused silica wafer (Figure 3). Initially, a 600-nm-thick amorphous silicon layer was deposited on the substrate via plasma-enhanced chemical vapor deposition (PECVD). Subsequently, chromium (Cr) was deposited on the amorphous silicon layer as a hard mask for etching the nanopillar arrays, and ensure compatibility with the DUV lithography system, which cannot recognize transparent substrates. Then, the intended metasurface patterns were transferred to a spin-coated photoresist layer using DUV projection lithography. Figure 3b presents a photograph of the wafer after photolithography. Next, nanopillars were formed by inductively coupled plasma (ICP) etching. Finally, the Cr hard mask was removed by wet etching and the wafer was diced into individual devices by blade dicing (Figure 3c). Scanning electron microscopy (SEM) characterization confirmed the effectiveness of the process (Figure 3d and e).

## 3 Results and discussions

### 3.1 Performance of the metasurface-based light receiver

The light receiver in a LiDAR system is a typical non-imaging optical system, with the FOV and the capability to enhance the received signal strength as two critical parameters affecting the detection range and SNR. The FOV defines the angular extent over which the system can receive echo signals, while the capability to enhance the received signal strength refers to the increased SNR and intensity of the signal captured by the receiver relative to a mere detector or other configurations.

**Figure 4:**
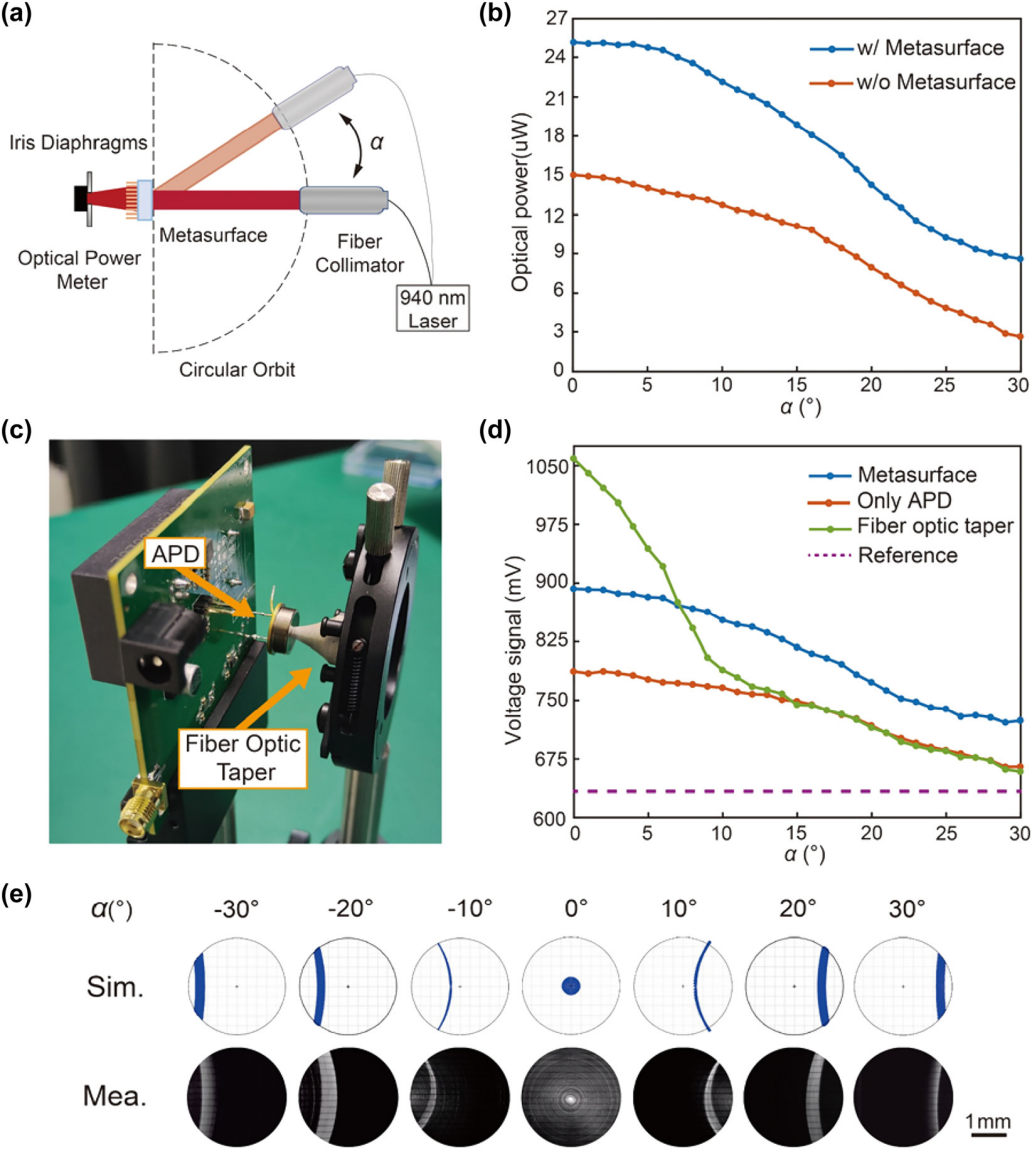
Experimental characterization of the metasurface-based light receiver. (a) Schematic of the experimental setup for assessing the light-gathering performance of the metasurface. *α* denotes the incidence angle. (b) Measured optical power of the light receiver upon various incident angles with/without the metasurface device. (c) Photograph of a practical light receiver including the avalanche photondiode (APD) and the fiber optic taper. (d) Measured voltages of the light receiver upon various incident angles of different configurations. (e) Measured (Mea.) and simulated (Sim.) light field intensity distributions at different incident angles.

These two parameters were experimentally characterized to assess the light-gathering performance of the proposed metasurface device, as shown in Figure 4. The experimental setup is exhibited in Figure 4a. The fiber collimator includes a collimating lens that produces a 20-mm-diameter beam and a 0.1 % light attenuator. An iris diaphragm is positioned in front of the sensor of the optical power meter, with the aperture size set to 3 mm to emulate the effective photosensitive area of the APD. Given the symmetric configuration, we only considered the incident angle from 0 to 30°. The measured results (Figure 4b) demonstrate a significant power enhancement ratio of approximately 1.5–3 times compared to the scenario without the metasurface. This non-uniformity may be further mitigated by more rational segmentation of the metasurface area. The decreasing trend of the received optical power originates from the uneven intensity distribution of the Gaussian beam, as well as the reduced optical power per unit area resulting from higher incident angles. Overall, the experiment results highlight the remarkable light-gathering ability of the fabricated metasurface across the targeted FOV of ±30°.

Similar measurements were conducted to evaluate the light-gathering performance of a practical light receiver, with an APD (Hamamatsu, S8890-30) sensor replacing the optical power and the iris diaphragm in Figure 4a. The APD can convert optical signals into electrical voltage biases. In addition to the metasurface, a fiber optic taper with a large-end diameter of 18 mm and a small-end diameter of 3 mm was also investigated for comparison, as shown in Figure 4c. The dotted reference line in Figure 4d indicates the voltage signal of the APD under dark conditions, which is closely associated with the circuit design. The results (Figure 4d) demonstrate that the fiber optic taper exhibits superior signal strength enhancement within a narrow range of incident angles but its performance degrades rapidly as the incident angle increases. In contrast, the metasurface-based light receiver displays a rather uniform performance across the entire FOV, resulting in a much larger detection range of the LiDAR system.

Furthermore, we utilized a CMOS image sensor to measure the light intensities on the receiving plane upon various incident angles (Figure 4e). It was observed that there is always a certain amount of light energy distributing within the central 3-mm-diameter circle area, enabling the large FOV feature of the metasurface-based receiver. The intensity distributions agree well with the simulated results obtained through ray-tracing techniques, thereby validating the design and fabrication of the metasurface device.

### 3.2 Application in a MEMS LiDAR system

**Figure 5:**
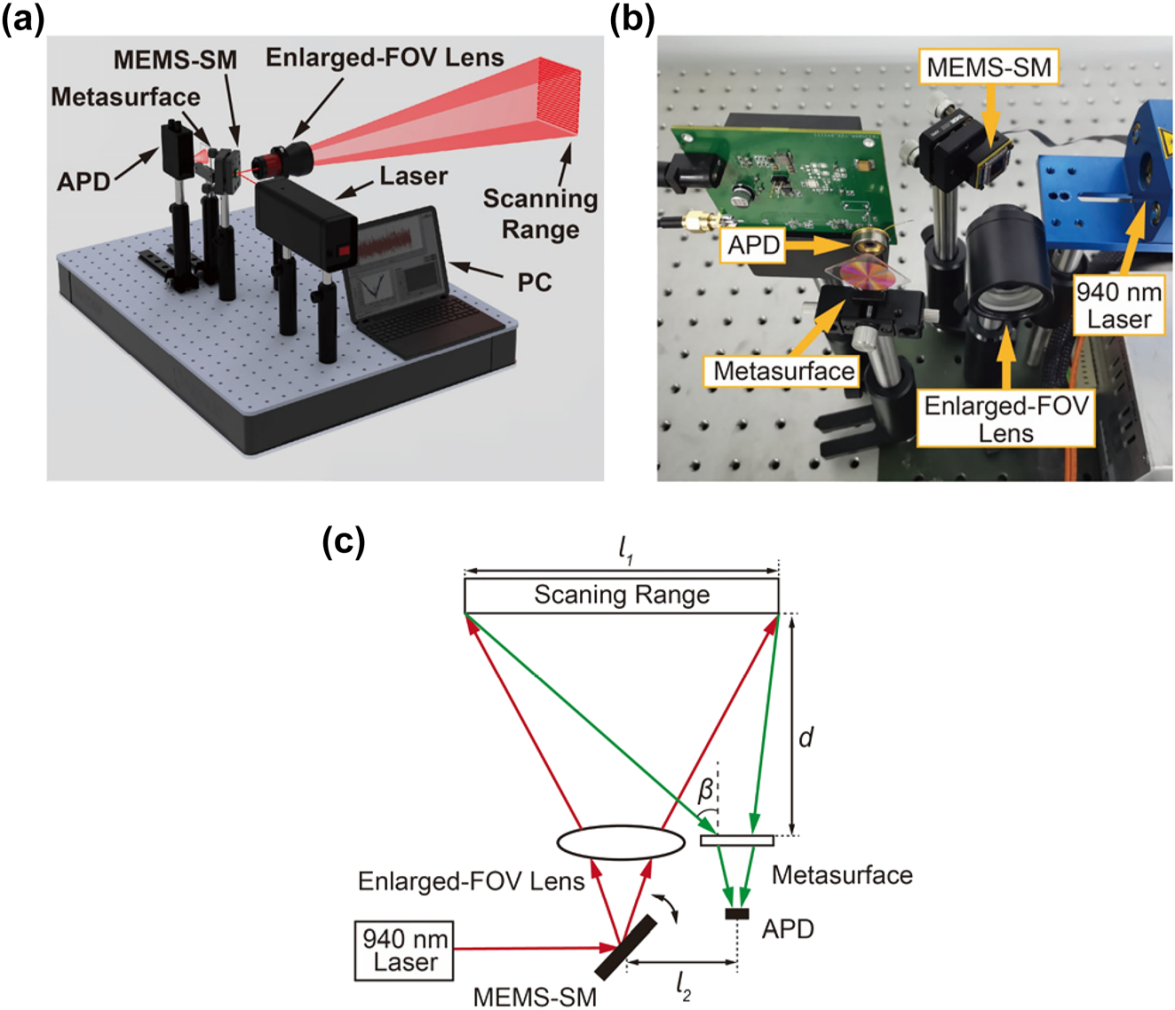
Experimental setup of the self-built LiDAR system. (a) Schematic diagram of the MEMS LiDAR system. (b) Photograph of the MEMS LiDAR system. (c) Geometrical illustration of the MEMS LiDAR system.

The proposed metasurface-based light receiver was subsequently integrated into a custom-built MEMS LiDAR system to demonstrate its applications in practical scenarios, as shown in Figure 5a and b. A modulated beam from a 940 nm laser source is incident on the MEMS scanning mirror (MEMS-SM) (Mirrorcle, S30348), which dynamically redirects the reflected beam towards an enlarged-FOV lens. By controlling the MEMS-SM, the output beam is deflected across a predefined scanning range. Then, the echo signals from the target are captured by a receiving system including the metasurface and the APD. The distance values are obtained by the time-of-flight (ToF) method, and data-processed 3D images are displayed on PC. Figure 5c illustrates the geometrical schematic of the MEMS LiDAR system. The maximum receiving angle *β* in the MEMS LiDAR system can be calculated as:

(6) $$\beta =\arctan\left(\frac{l_{1}+2l_{2}}{2d}\right)$$

where *l*_1_ denotes the scanning range length including the target and background, *l*_2_ represents the distance between the MEMS-SM and the APD, and *d* is the distance between the target and metasurface. When *l*_1_ and *d* are fixed, we can obtain different *β* values by varying *l*_2_.

**Figure 6:**
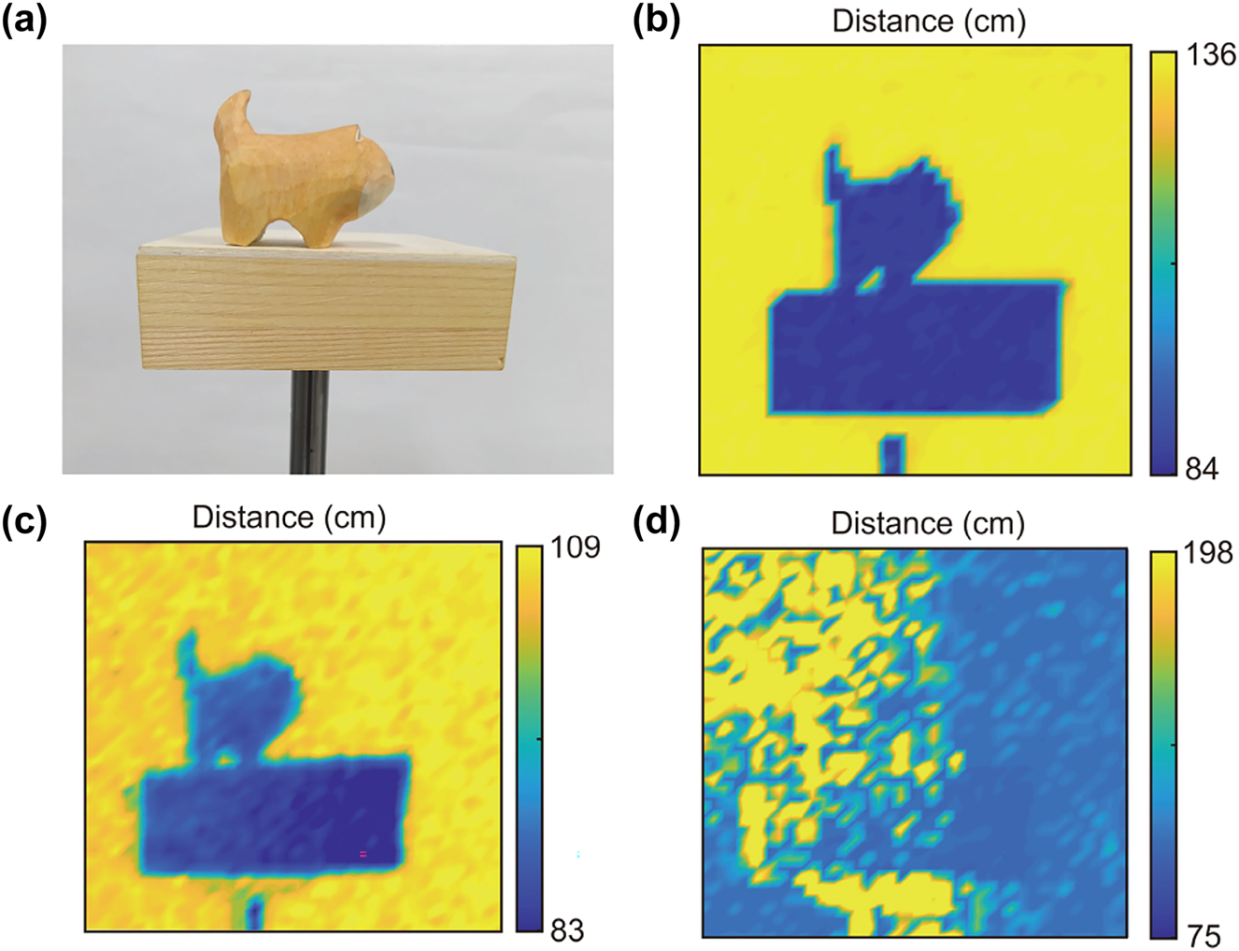
3D imaging performance of the MEMS LiDAR system. (a) Photograph of the target for imaging. (b) Depth map obtained by the system in a small FOV with the metasurface-based light receiver. (c, d) Depth maps obtained by the system in a large FOV with the metasurface-based receiver and fiber-optic-taper-based receiver, respectively.

To investigate the large FOV 3D imaging capability of the MEMS LiDAR, a wooden dog model (45 × 25 × 35 mm) standing on a wooden pedestal (85 × 85 × 27 mm) was selected for depth profiling, a paper baffle located at a certain distance from the scanning target was used as the background, as shown in Figure 6a. A grid of 40 × 40 equally distributed points along the scanning path are utilized for distance retrieving. The distance *d* was fixed at 84 cm, the scanning range was about 13 × 13° and the length *l*_1_ was 12 cm. The distance *l*_2_ was first set to 3 cm with the maximum receiving angle *β* at 7.5°. The obtained depth map of the target is exhibited in Figure 6b. The outlines of the wooden dog model and the wooden pedestal are clearly distinguished, and the distance precision is evaluated to be less than 2 cm. This experiment validates the light-gathering ability of the metasurface-based light receiver in a small FOV.

Next, the distance *l*_2_ was changed to 42 cm, resulting in a maximum receiving angle *β* of 30°. 3D imaging using the metasurface or fiber optic taper as the receiving optics is conducted for comparisons, and the results are displayed in Figure 6c and d, respectively. These experiments were conducted within a FOV ranging from 23° to 30°, which is detailed in Section S2 (Supplementary Material). The former image retains the details of the objects and the background, whereas the latter image is severely blurred and fails to resolve most information about the objects. These experimental results agree well with Figure 4d and validate the metasurface’s ability to effectively receive the echo light signals at an incident angle as large as ±30°. The imaging experiment underscores the extraordinary potential of the metasurface for use in the compact and highly integrated LiDAR systems.

## 4 Conclusions

This study systematically investigates the metasurface-based light receiver, establishes a general design methodology based on mapping relationships, and experimentally validates its performance in a MEMS LiDAR system. The methodology establishes a design framework that correlates metasurface parameters with APD diameter, system dimensions, and FOV requirements. By employing a segmented phase compensation strategy and DUV lithography-based mass production, we demonstrated a compact and cost-effective solution that a 20-mm-diameter 4-region metasurface device achieves a significant signal enhancement ranging from 1.5 to 3 times across ±30° FOV. Furthermore, integration into a MEMS LiDAR system confirms its feasibility for real-world applications, delivering clear 3D imaging even at wide angles, thereby verifying system-level feasibility. This innovative approach not only reduces the size and weight of the LiDAR system but also ensures high performance, making it a promising solution for various applications such as UAVs, miniaturized robots, and self-driving cars. Future research efforts may focus on refining the design of mapping relationships for metasurface devices to achieve higher enhancement and greater uniformity in performance.

## Supplementary Material

Supplementary Material Details
