# Supplementary material for: Metasurface-based large field-of-view light receiver for enhanced LiDAR systems
Source: Nanophotonics. 2025 Jul 2;14(15):2673–82. doi: 10.1515/nanoph-2025-0161 (PMC12322719; doi:10.1515/nanoph-2025-0161)
Supplement: Supplementary file 1 — Supplementary Material Details [file j_nanoph-2025-0161_suppl_001.docx]

**Supplementary Material**

**Table of Contents Section**

**Section S1.** The performance of cylindrical meta-atoms under various incident angles.

**Section S2.** The specific receiving angle range for the 3D imaging experiment.

**Section S1. The performance of cylindrical meta-atoms under various incident angles.**

It is notable that, in the case of a large FOV, the complex transmission coefficients of the meta-atoms at different incidence angles must be taken into account. To verify the incident-angle-insensitivity of the cylindrical meta-atoms, additional numerical simulations were performed, and the results are presented in Fig. S1. Fig. S1a demonstrates the optical power transmission of the meta-atoms at various incident angles. Fig. S1b illustrates the phase delay values of the meta-atoms relative to the first meta-atom (*D* = 160 nm) at different incident angles. Fig. S1c shows the deviation in the phase of the meta-atoms under oblique incidence compared to normal incidence. The results indicate minimal phase and transmission variations within the ±30° FOV. By optimizing the aspect ratio of the cylindrical meta-atoms, the insensitivity to the incident angle can be further improved, as illustrated by the previous works [S[1](#_ENREF_1), S[2](#_ENREF_2)].


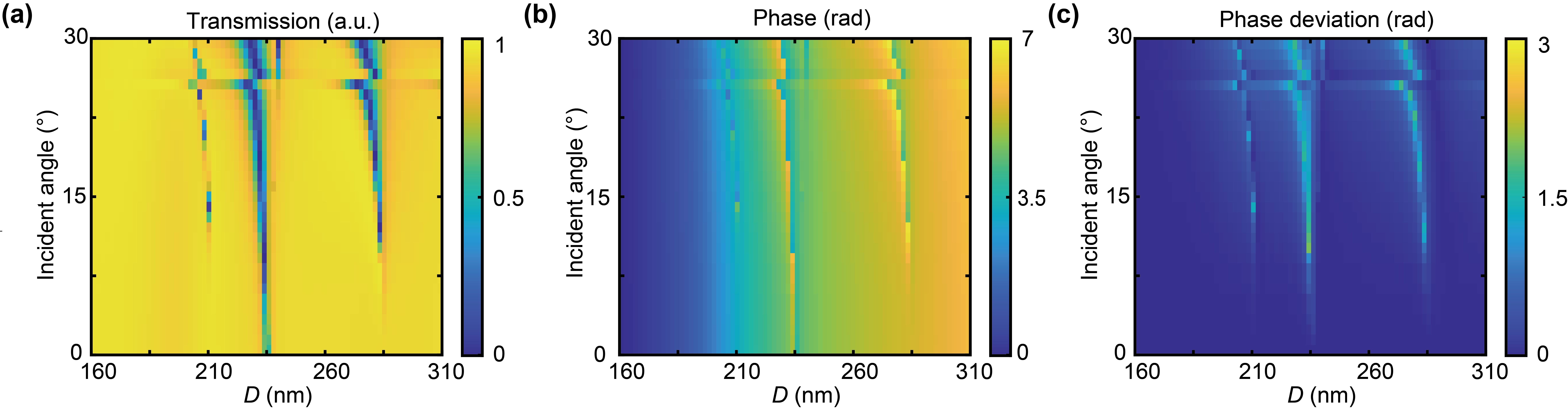


**Fig. S1** | The performance of cylindrical meta-atoms under various incident angles. (a) Angle-dependent optical power transmission and (b) phase delay values of the cylindrical meta-atoms with *H* = 600 nm and varying *D* values ranging from 160 to 310 nm. (c) The deviation in the phase of the meta-atoms under oblique incidence compared to normal incidence.

**Section S2. The specific** **receiving angle range for the 3D imaging experiment.**

Due to the limitation of the scanning range in the transmitting system, the 3D imaging results display only a portion of the receiving FOV. Fig. 6b shows the receiving capability of the metasurface-based receiver within a FOV less than 7.5°. Fig. S2 illustrates the geometrical schematic of the receiving angle range in Fig. 6c and 6d. According to Eq. (6), the maximum receiving angle *β*_1_ and the minimum receiving angle *β*_2_ can be expressed as:

$$\left\{\begin{matrix} \beta_{1}=\arctan\left(\frac{2l_{2}+l_{1}}{2d}\right) \\ \beta_{2}=\arctan\left(\frac{2l_{2}-l_{1}}{2d}\right) \end{matrix}\right.$$

which *l_1_* denotes the scanning range length including the target and background, *l_2_* represents the distance between the MEMS-SM and the APD, and *d* is the distance between the target and metasurface. Based on the values of *l_1_*, *l_2_* and *d* presented in Section 3.2, the maximum receiving angle *β*_1_ and the minimum receiving angle *β*_2_ is calculated to be approximately 30° and 23°. Fig. 6c and 6d show the receiving capability of the metasurface-based receiver and the fiber-optic-taper-based receiver within a FOV of 23°~30°. The comparison between the results in Fig. 6c and 6d demonstrates the superior performance of the metasurface-based receiver in a large FOV.


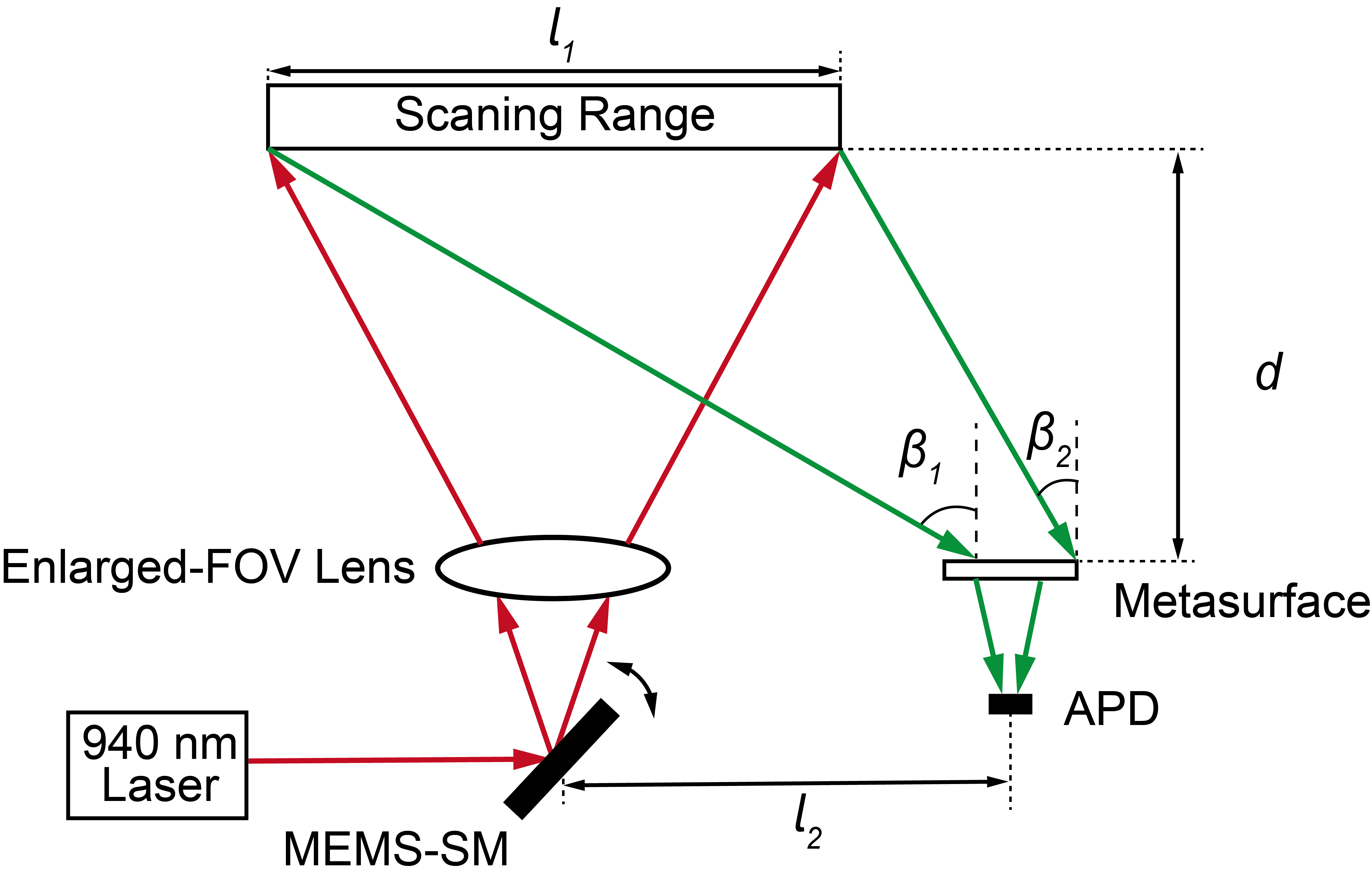


**Fig. S2** | Geometric illustration of the receiving angle range in Fig. 6c and 6d.

**References:**

[S1] W. Liu *et al.*, "Metasurface Enabled Wide-Angle Fourier Lens," *Adv. Mater.,* vol. 30, no. 23, p. 1706368, 2018.

[S2] H. Zheng *et al.*, "Compound Metalens Enabling Distortion-Free Imaging," *Engineering,* vol. 45, pp. 52-58, 2025.
